# Successful live birth in a woman with resistant ovary syndrome treated with letrozole and HMG: A case report

**DOI:** 10.1097/MD.0000000000020199

**Published:** 2020-05-15

**Authors:** Zhenni Mu, Jingyan Song, Yi Yu, Zhengao Sun

**Affiliations:** a Traditional Chinese Medicine Institute, Shandong University of Traditional Chinese Medicine; b Reproductive and Genetic Center of Integrated Traditional and Western Medicine, Affiliated Hospital of Shandong University of Traditional Chinese Medicine, Shandong Province; c The First Clinical College, Shandong University of Traditional Chinese Medicine, Jinan, China.

**Keywords:** letrozole, ovulation induction, Resistance ovary syndrome

## Abstract

**Introduction:**

Resistance ovary syndrome (ROS) is a disease characterized by hypergonadotropic amenorrhea but with normal ovarian reserve. Currently, its pathogenesis is still unclear and the treatment methods are complex. Nevertheless, there are evident negative effects of this disease on females’ physical and mental health such as gonadal dysplasia, infertility, anxiety, and depression. This article reports a case of successful ovulation induction and pregnancy with letrozole combined with HMG. This can provide clinical treatment guidelines for the disease.

**Patient concerns:**

The patient underwent several hormone replacement cycles and ovulation induction cycles. But the dominant follicles were not extracted even after using large doses of gonadotropin.

**Diagnosis:**

Resistant ovary syndrome; Primary infertility

**Interventions:**

Larger doses of letrozole combined with HMG were injected to stimulate ovulation and sensitize the ovaries during menstruation. This helped to examine the peripheral effects of letrozole in relation to gonadotropin.

**Outcomes:**

The patient displayed a dominant follicular growth and notable ovulation which resulted in a full-term pregnancy and successful delivery.

**Conclusions:**

The resistance ovary syndrome (ROS) can be treated and the findings from this case provides a possible treatment for ROS patients with infertility.

## 1 Introduction

Resistance Ovary Syndrome (ROS)/ Insensitive Ovary Syndrome (IOS)/Savage Syndrome is a disease characterized by high endogenous gonadotropin levels, low estrogen levels and normal ovarian reserve. Most patients show reduced responses to exogenous gonadotropin. Generally, ROS is a rare clinical condition considered as a subtype or specific type of premature ovarian failure and it affects approximately 11% to 20% of patients with hypergonadotropic amenorrhea.^[1]^ The ROS has low incidence rates and evidence on its pathogenesis and treatment methods is limited. Investigating the effects of stimulating the ovulation process is therefore important in developing treatment for infertility. Here, we describe a case of a woman with fertility problems due to ROS, and the oviduct factors was excluded. She underwent several cycles of estrogen and progesterone replacement therapy, HMG ovulation induction therapy and controlled hyperstimulation. There was no evident dominant follicular development after down-regulating the oviduct factors. The woman successfully ovulated and conceived after treatments with Letrozole (FuRui, Hengrui Medicine, China) combined with HMG (menotrophin, Livzon Pharmaceutical Group Inc, China). She eventually delivered a full-term healthy baby.

## 2 Case report

The patient was a 31-year-old woman with a history of infertility for 6 years. Her BMI was 22.17 kg/m^2^ and she had experienced irregular menstrual cycles with intervals of 30 to 180 days. She was put on hormone replacement and oral contraceptive therapy to regularize her menstruation. After withdrawal of the therapies, her menstruation cycle would commence normally. She was admitted at the center for primary infertility in September 2014. Gynecological examinations showed that she had unusual pubic hair pattern, pale labia minora, narrowed cervix, small uterus, and low vaginal secretions which limited the entry of speculum. An ultrasonogram showed the woman had a smaller uterus, ovaries and an antral follicle number of 5 to 6. Hysterosalpingography (HSG) revealed that the bilateral fallopian tubes were unobstructed. Laboratory examinations showed the following hormonal concentrations in serum: anti-Müllerian hormone (AMH), 4.37 ng/ml; basic follicle stimulation hormone (FSH), 21.83 U/L; luteinizing hormone (LH), 14.08 U/L; estradiol (E_2_), 12 pg/ml; prolactin (PRL), 28.22 ng/ml; testosterone (T), 0.26 ng/Ml.; and thyroid stimulating hormone (TSH), 0.29 mIU/L. A peripheral chromosomal examination displayed a 46, XX karyotype. The patient's spouse was 31 years old and had no smoking and alcohol history. His peripheral chromosomal examination displayed a 46, XY karyotype. She was therefore initially diagnosed with primary infertility and resistant ovary syndrome (ROS).

### 2.1 October 2014 to November2014

In October 2014, the patient received the first cycle of ovulation induction treatment at our center. The HMG (150 IU) was injected intramuscularly on the fifth day of menstruation and 75 IU was added every 3 days. The ultrasonogram monitored the follicular development. The follicles did not develop until the dosage of HMG was increased to 450 IU. The dose of HMG was maintained and the follicular development was monitored by ultrasonogram continuously. When the follicular diameter reached 14 mm, the growth was inhibited by continuous medication. The HMG (6075 IU) and chorionic gonadotropin (HCG, Livzon Pharmaceutical Group Inc, China) (10,000 IU) were used. The medications were injected intramuscularly to induce oocyte maturation. Despite having sexual intercourse at the anticipated time, the patient did not conceive.

### 2.2 December 2014 to March 2017

After the ovulation induction failed, the patient was treated intermittently with estrogen and progesterone replacement therapy to maintain the menstruation cycle every 2 to 3 months. In May 2015, the patient underwent HSG treatment for the second time when it was suggested that the right fallopian tube was incompletely obstructed and the left fallopian tube was unobstructed. The patient qualified to undergo assisted reproductive technology (ART) and in vitro fertilization (IVF) technology to induce conception after medical tests indicated that she had a tubal factor and ovulation disorder. The patient underwent gonadotrophin releasing hormone antagonists (GnRH-ants) treatment protocol. Before the ovulation induction, a circle of oral contraceptives (Yasmin, Bayer, Germany) was used to adjust the internal secretions. The serum hormone concentrations on the third day of menstruation after withdrawal were as follows: FSH 17.17 U/L, LH 8.85 U/L, E_2_ 13 pg/ml. We administered a combination therapy of HMG 225 IU, Recombinant Human Growth Hormone for Injection (rhGH, Shanghai United Cell Biotechnology Co., Ltd, China) 4 IU and estradiol valerate tablets (E_2_V, Bayer, Germany) 1 mg, and the dosage of HMG was increased every 4 days. The drug was used for 12 days and the total amount of HMG was 3600 IU, rhGH was 48U and E_2_V, was 12 mg. The E_2_ levels were 42, 54, and 70 pg/ml on day 4, 8, and 12, respectively. The ultrasonogram indicated that the largest follicular diameter in bilateral ovaries was 6 mm, and there was no dominant follicular development. There was continued use of HMG 450U for 12 days combined with clomiphene citrate capsules (CC, Shanghai HengShan Pharmceutical CO., LTD, China) 150 mg for 5 days. The E_2_ levels were 100, 60, and 123pg/ml on day 16, 20, and 24, respectively. The ultrasonogram showed that bilateral ovaries had no evident dominant follicular development then we abandoned the cycle (Table 1).

**Table 1:**
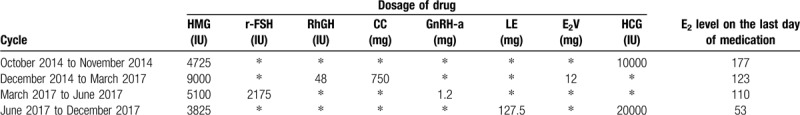
The dosage of drug in different cycles.

### 2.3 March 2017 to June 2017

Since there was no dominant follicular development in the previous ovulation induction cycle, we adopted the long-term protocol in this cycle. On the 4th day of menstruation, we injected her with 1.2 mg of Beiyi (3.75 mg of Leuprorelin Acetate Microspheres, Livzon Pharmaceutical Group Inc, China). After 28 days i.e., the 32nd day of menstruation (Gn starting day), we observed the following hormone concentrations in serum: FSH 4.34 U/L, LH 1.92 U/L, E_2_ 11 pg/ml. We used r-FSH (Gonal-F, Merck, Germany) 75 IU, HMG 225 IU as initial dose, then the further dosage was increased/decreased accordingly. The drug was used for 18 days and the total amount of HMG was 5100 IU and rFSH was 2175 IU. The E_2_ levels were 28, 65, and 110 pg/ml on day 6, 12, and 18, respectively. The ultrasonogram indicated that the largest follicular diameter in bilateral ovaries was 6 mm, and there was no evident dominant follicular development. Therefore, we decided to abandon the cycle.

### 2.4 June 2017 to December 2017

**Figure 1:**
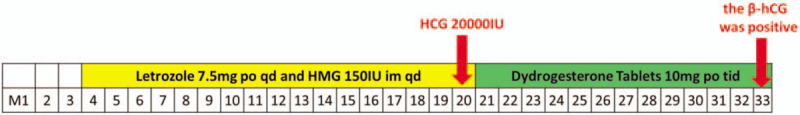
The successful cycle of ovulation induction therapy.

Due to the repeated ovulation induction failures, the patient's low sensitivity to Gn was a suspected cause, thus high-doses of Gn were no longer used for ovulation induction. A new cycle of ovulation induction therapy was performed after 3 cycles of hormone replacement therapy considering that Letrozole could promote ovarian sensitivity to gonadotropin in the periphery. We observed the following hormone concentrations in serum on the fourth day of menstruation: FSH 21.66 U/L, LH 13.75 U/L, E_2_ 19 pg/ml. The patient was given Letrozole 7.5 mg combined with HMG 225 IU for ovulation induction, and the follicular development was monitored regularly. We discovered that the left ovary had slow follicular growth after maintaining the dose. On the 17th day of treatment, ultrasonogram showed that the largest diameter of the left ovarian follicle was 17 mm, while the right ovary had no dominant follicle development; and the following hormone concentrations in serum: LH 34.22 U/L, E_2_ 53 pg/ml, P 1.69 ng/ml. At that time, E_2_ levels were very low, but the LH and P levels were higher than their baseline values. Since several literature concluded that letrozole can inhibit the production of estrogen, the E_2_ levels do not clearly reflect follicular development. The HCG 20000 IU was injected on the same day whilst referring to LH and P levels, then patients were instructed to do sexual intercourse on the same day and the next day. The ultrasonogram showed follicular excretion after 3 days. The patient was given luteal support with dydrogesterone tablets (Duphaston, Solvay Pharmaceuticals B.V, Netherlands) 10 mg po bid. The serum beta-human chorionic gonadotropin (β-hCG) was positive (348 U/L) 2 weeks later (Fig. 1). On the 59th day of menstruation, the ultrasonogram showed the following: intrauterine early pregnancy (7 weeks of gestation) and a fetal heartbeat of 131 beats per minute. Subsequently, the patient underwent various prenatal examinations regularly, and all the indicators were normal. After an uneventful 39-week and 2 days pregnancy, the patient spontaneously delivered a healthy boy of 3005 g.

## 3 Discussion

Jones and Ruehsen^[1]^ were the first to discover and report about ROS in 1967. The patients with ROS have high gonadotropin serum levels, low estrogen serum levels and normal ovarian reserve (normal AMH, statin B levels and normal amounts of primordial follicles). Currently, clinical studies have found that the pathogenesis of ROS may have correlation with the inactivation mutation of FSH, the abnormal signal transduction of Gn in ovarian membrane, the lack of follicle-stimulating factors, the mutation or defect of FSH receptor gene, the abnormal regulation of granulosa cell proliferation factor, etc.

In our ROS patients, sufficient HMG were used in the first cycle of ovulation induction, but the follicular development stooped at 14 mm, which may due to the increase of 3’, 5’ - cyclic adenosine monophosphate (cAMP) level in ROS patients after using large dose of HMG. On the one hand, it promoted the growth of primordial follicles,^[2]^ while on the other hand, it inhibited the maturation of oocytes.^[3]^ Since the first ovulation cycle only obtained follicular development but not follicular maturation, we considered that the patients may not simply have a follicular development disorder caused by Gn deficiency. We found that rhGH could promote follicular growth, steroid hormone synthesis, and enhance ovarian response to FSH,^[4]^ though there are no successful cases of rhGH stimulation in ROS patients. Therefore, in the second cycle, we tried to add rhGH 4 IU/d on the basis of HMG, but the patient had no dominant follicular development, which was in line with the results reported by Mueller.^[5]^ Since the bilateral ovaries were still in the basal state at this time, clomiphene 150 mg/d was added for 5 days to collect follicles, but still no dominant follicular development. The mechanism of clomiphene is to occupy the receptor of estrogen without the effect of estrogen, and its negative feedback induces the hypothalamic-pituitary-ovary axis to promote follicular development and ovulation.^[6]^ In such patients, the development of non-dominant follicles may be caused by the decreasing sensitivity of pituitary GnRH receptors resulting from long-term higher level of basal FSH and LH. Thus, in the third cycle, GnRH-a was used for down-regulation in order to increase the sensitivity of hypophysis, and r-FSH was then added for ovulation induction, but unfortunately, there was still no dominant follicular development.

The successful ovulation induction is primarily determined by Gns ability to act effectively on the ovarian receptors and promote follicular growth. The repeated use of large doses of Gn for ovulation induction did cause superior follicular development, suggesting that the ovulation disorder caused by Gn deficiency was not only due to the Gn deficiency, but may also due to the lack of Gn receptor on ovarian surface, the absence of some cytokines promoting follicular development, the abnormal Gn signal transduction on ovarian surface etc. This factor should be considered when performing ovulation induction procedures. Thus, the high-dose Gn can treat the condition as well as increasing the patients’ sensitivity to Gn. Therefore, in the fourth cycle, we chose letrozole in combination with HMG for ovulation promotion and the patient successfully got pregnancy and live birth. Letrozole is the third-generation aromatase inhibitor. Its’ peripheral effect is vital for a successful ovulation induction in ROS patients. In the peripheral ovarian tissues, letrozole inhibits the conversion of androgen to estrogen by inhibiting aromatase activity, resulting in a transient accumulation of androgen. The accumulated androgen stimulates expression of insulin-like growth factor I as well as other autocrine and paracrine factors, thereby increasing the ovarian response to gonadotropins.^[7]^ When combined with HMG, this maximizes the effects and using small doses of HMG effectively stimulates ovulation simultaneously reducing the costs. Since letrozole inhibits estrogen production, ultrasonogram follicular size, serum LH and P should be used to determine the duration of HCG administration.

Formulating an appropriate ovulation induction program and choosing the right ovulation induction drug is essential to follicular development in ROS patients. Letrozole combined with HMG is a fast and effective method for ovulation induction in ROS patients. The letrozole dose is usually higher than the normal ovulation dose, generally starting at 7.5 mg. The initial HMG dose is normally 225 IU, which is used continuously until the dominant follicle develops. When compared to other ROS treatments such as IVM,^[8]^ COS,^[9]^ the treatment used in this study is affordable and the success rate was higher. This confirms that our treatment is effective for ROS patients with fertility problems. Nevertheless, future investigations should examine the clinical effects of the present treatment.

## Author contributions

**Data curation:** Zhengao Sun.

**Supervision:** Jingyan Song, Zhengao Sun.

**Writing – original draft:** zhenni Mu.

**Writing – review & editing:** Jingyan Song, Yi Yu, Zhengao Sun.
